# Topics of Public Interest

**Published:** 1915-09

**Authors:** 


### TOPICS OF PUBLIC INTEREST.

---

**Warning.** Dr. H. W. Humphrey of Lowville, N. Y., warns against a man claiming to represent the "International Automobile League" with headquarters at Buffalo, and selling stock, membership, etc.

---

**Population of Buffalo.** The state census gives a population of 459,508, an increase of 8.4 per cent. since 1900.

---

**Drug Thefts.** Dr. Burt J. Maycock of Buffalo and others have reported thefts of morphine, etc., by waiting patients. The difficulty of securing narcotics will undoubtedly require greater caution than usual against thefts of this kind.

---

**Insanity Increasing.** 36,240 inmates of public and private asylums, are reported by the Secretary of State of N. Y. The ratio per 1,000 population has steadily increased as follows: 1890, 249; 1900, 304; 1912, 329.7; 1915, 352.6.

---

**Proposed Reduction of State Boards.** The State Charities

Aid Assn. opposes the plan of reducing the control of all charitable and penal institutions of the state to a single board and denies the allegation that such institutions are at present under the control of 26 different boards. Three boards have been discontinued, two others have no relations to philanthropic institutions, seven "have the same or substantially the same relations to state institutions as to all departments of the government and these relations will probably always of necessity continue"; four are expert bodies with powers of inspection and recommendation as to particular features, five more have similar powers. "Simmered down, there are actually four officials or commissions that have any actual powers of control of state charitable institutions." These are: the fiscal supervisor, the sites buildings and grounds commission, the buildings improvement commission, the salary classification commission.

---

**Special Requirements for Health Officers.** After November 1, 1916, local health officers appointed in New York State will be subject to the following requirements: medical degree obtained at least three years previously; between 24 and 65 years of age; special training consisting (a) of one year correspondence course with one week resident laboratory and field work, under control of State Public Health Council; or (b) approved 6-weeks course in Public Health at some educational institution; or (b) evidence of substantial compliance with foregoing requirements. These requirements may be waived by the Public Health Council under special conditions specified by the local board of health. These requirements affect about 1,200 officials. Appointees prior to November 1, 1916 will be made to agree to conform to the requirements within the first year of office.

---

**Narcotics Law. Important Decision Secured.** On the 28th day of July, 1915, Hon. Philip A. Laing, County Judge of Erie County, handed down a decision "In the Matter of the Petition of John W. Kales, for an order to return his private papers, books and other property forcibly seized on his premises, 139 South Division Street, Buffalo, N. Y., by Wesley C. Dudley, as district attorney, and his assistant, Herbert B. Lee, on or about May 20th, 1915." The decision of said County Judge directs the district attorney to return to said Kales or his attorney, Alfred L. Harrison, on service of the order, the record book containing the names and addresses of the patients treated by said Kales with morphine and any and all other property which said district attorney or his agents removed from the premises of said Kales on said May 20th. The same have been returned.

This decision was rendered in consequence of a petition made by said Kales on June 7th, 1915, and argued before said County Judge on June 8th, 1915, at which time of argument the district attorney returned in Court: One bottle filled with morphine, part of a hypodermic syringe, a small box containing one eighth of a grain of morphine, an empty bottle formerly containing morphine tablets and one or two other little articles which the district attorney's assistant seized on said day. But the district attorney insisted on retaining the record book containing the names and addresses of the patients treated by said Kales with morphine.

Mr. Harrison, the attorney for said Kales, at said argument, maintained that the seizure was illegal and lawless as it was in violation of the Constitution of the United States and the Constitution of the State of New York on two grounds, namely: (1) That no one shall be compelled to be a witness against himself in a criminal manner; nor shall any one be deprived of his liberty or property without due process of law; and (2) The right of people to be secured in their persons, houses, papers and effects, and that no warrant shall issue therefor except for probable cause supported by oath or affirmation particularly describing the place to be searched and the persons or things to be seized.

In the case of Dr. Kales, by seizing his own books and papers, he was being compelled to be a witness against himself. The Court in a leading United States case says:

"Individual may stand upon his Constitutional rights as a citizen. He is entitled to carry on his private business in his own way. His power to contract is unlimited. He owes no duty to the State or to his neighbors to divulge his private business or to open his doors to an investigation, so far as it may intend to incriminate him."

And the Court, further defining the words "due process of law" says it means, "Law in its regular course of administration through the Courts of Law." And "process" means, "One issued by **judicial** officer."

This decision of Judge Laing holds and emphasizes the fact that the record book required by the law to be kept at the office of the physician cannot, and no other property, can be removed by the district attorney or any other officer, except through due process of law, namely, by an order of the Court properly obtained.

---

**Ward for Drug Habitues.** An appropriation of $2000 has been made for equipping a ward in the Buffalo Municipal Hospital on E. Ferry Street and for paying for the care of patients. 30 can be accommodated at once.

**Playground for Batavia.** The grounds at Main and Jefferson Streets were formally opened August 6.

---

**Failure to Report Births** within 5 days, as required by law in N. Y. State, will subject physicians to prosecution by the local district attorneys.

---

**A. M. A. Attendance.** The California State Journal of Medicine claims that 1054 members of the State Society—more than 50% of the total—attended the recent A. M. A. meeting in San Francisco, the total registration being a little over 2300. Apparently the out-of-town attendance at the A. M. A. was relatively less and absolutely only slightly greater than at the State meeting in Buffalo.

---

**An Open Air School** will be established in Hornell, this fall.

---

**Typhoid.** On August 18, 12 cases were reported to the Buffalo Department of Health, the largest number ever reported in a day. This statement has been made in the daily press. Dr. Gram authorizes us to deny it and to state that there has been no unusual number of cases and that a large share of those reported have been patients brought into the city or residents who have contracted the infection elsewhere.

---

**The Buffalo Industrial Show** will be held afternoons and evenings, September 22-Oct. 2; admission 25 cents. Physicians are asked to attend, not only because a prosperous business community means a prosperous profession, but because many of the exhibits will be along lines in which physicians are interested.

